# Enhancing Diagnosis: The Role of Point-of-Care Ultrasound in Early Detection of Pericardial Effusion in Post-Radioactive Iodine Hypothyroidism

**DOI:** 10.7759/cureus.73217

**Published:** 2024-11-07

**Authors:** Mae Jane Khaw, Wei Ven Chin

**Affiliations:** 1 Department of Medicine, Limbang Hospital, Limbang, MYS; 2 Department of Medicine, Sarawak General Hospital, Kuching, MYS

**Keywords:** case report, hypothyroidism, pericardial effusion, point-of-care-ultrasound, post radioactive iodine therapy

## Abstract

Pericardial effusion is a relatively common classical pericardial syndrome that poses a diagnostic challenge for clinicians. There are varying clinical presentations of pericardial effusion, ranging from asymptomatic incidental findings on chest X-ray (CXR) or point-of-care ultrasound (POCUS) to hemodynamic instability in cardiac tamponade. Pericardial effusion is a notable cardiac manifestation of severe and long-standing hypothyroidism. We report the case of a 57-year-old female with a previous history of Graves’ disease treated with radioactive iodine (RAI) more than 30 years prior but who had never been followed up on post-RAI. Admission CXR depicted a water bottle-shaped cardiomegaly. POCUS portrayed a massive pericardial effusion with no echocardiographic evidence of cardiac tamponade. Laboratory investigations showed raised thyroid-stimulating hormone and low free thyroxine. The pericardial effusion gradually subsided during the surveillance echocardiogram after initiation of thyroid hormone replacement therapy. Early POCUS assessments in this case expedite the diagnosis and management of hypothyroidism-induced pericardial effusion. With timely and adequate thyroid hormone replacement therapy, pericardial effusion can be reversed, thereby averting the fatal complications of cardiac tamponade.

## Introduction

Pericardial effusion is a relatively prevalent classical pericardial syndrome in clinical practice. There are myriad clinical presentations of pericardial effusion, ranging from asymptomatic incidental findings on chest X-ray (CXR) or point-of-care ultrasound (POCUS) to hemodynamic instability in cardiac tamponade. In normal human physiology, the pericardial cavity contains 10-50 mL of pericardial fluid as a plasma ultrafiltrate that serves as a lubricant between the pericardial layers. The speed of pericardial fluid accumulation greatly contributes to the hemodynamic impact of the pericardial effusion [1].

The majority of cases are idiopathic, accounting for up to 50% of all cases in developed countries. Other possible etiologies include infections (viral, bacterial, especially tuberculosis), neoplastic, autoimmune, cardiac (pericarditis, myocarditis, and heart failure), metabolic (such as hypothyroidism or renal failure), and drug-related (e.g., minoxidil) [2]. Hypothyroidism is an endocrine disorder that may result in a wide range of cardiac manifestations, such as pericardial effusion, bradycardia, conduction abnormalities, diastolic hypertension, and accelerated coronary artery disease [3]. Pericardial effusion in hypothyroidism can be attributed to the increased permeability of the epicardial vessels and decreased lymphatic drainage of albumin, which increases the colloid osmotic pressure and reduces the pressure gradient (between the pericardium and the pericardial space), resulting in fluid accumulation within the pericardial space according to Starling’s law [4]. The likelihood of this complication increases as the severity of hypothyroidism rises; mild cases have a 3-6% occurrence rate, whereas severe cases may experience pericardial effusion in 30-80% of instances [5].

## Case presentation

We report the case of a 57-year-old female with a previous history of Graves’ disease treated with radioactive iodine (RAI) more than 30 years prior. She presented with a two-day history of headaches and nausea. Upon arrival at the emergency department, she was hypertensive, with a blood pressure of 236/123 mmHg and a pulse rate of 63 beats per minute. Cardiac auscultation revealed muffled heart sounds with no murmur. Neurological examinations were unremarkable. Apart from the hoarseness of voice, none of the other systemic examinations were noteworthy.

**Figure 1:**
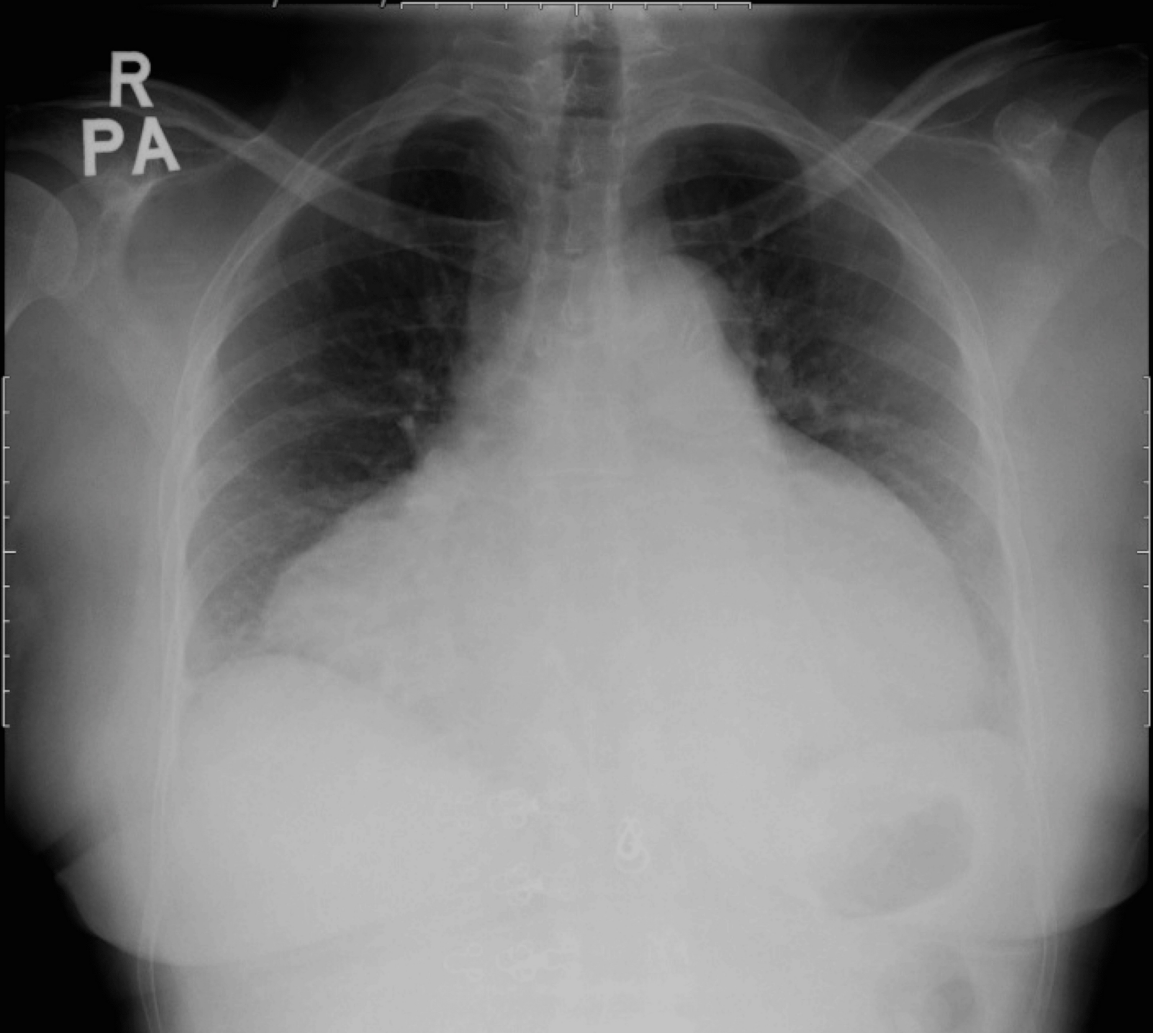
CXR on initial presentation demonstrating globular enlargement of the cardiac silhouette, resembling a water bottle configuration CXR: chest X-ray

**Figure 2:**
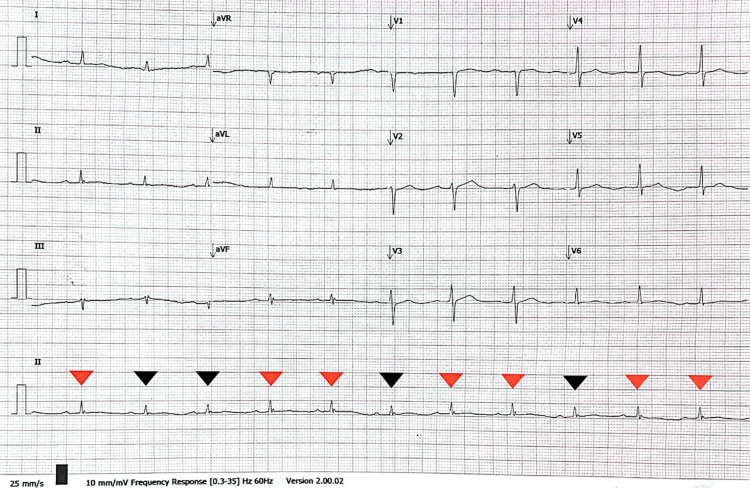
Electrocardiogram revealing low QRS voltage accompanied by electrical alternans, indicated by alternating QRS amplitudes in leads II, V1, V5, and V6, as illustrated by the red and black small triangles in lead II

Further exploration of her medical history revealed that she never had her follow-up thyroid function test post-RAI for nearly 30 years. In addition, she had experienced gradual worsening of effort tolerance for three years, as well as intermittent cold intolerance and voice hoarseness for the past 15 years. She denied other autoimmune symptoms or chronic cough. The admission CXR depicted a water bottle-shaped cardiomegaly (Figure 1), and the electrocardiogram showed a low-voltage QRS complex with electrical alternans (Figure 2).

**Figure 3:**
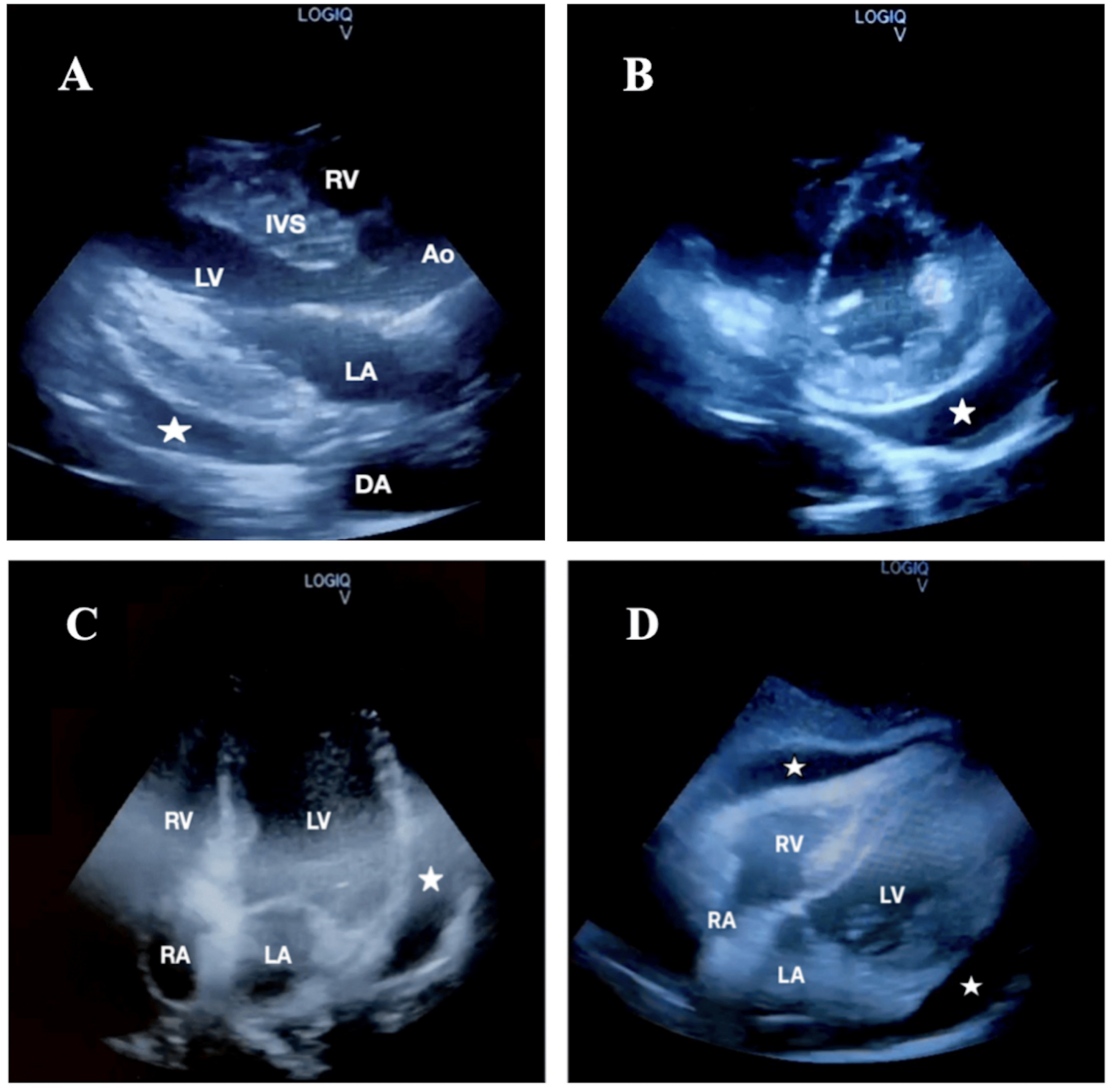
Standard echocardiographic views demonstrating pericardial effusion, indicated by the white star: (A) Parasternal long axis; (B) Parasternal short axis; (C) Apical four-chamber view; (D) Subcostal view Ao: aorta; DA: descending aorta; IVS: interventricular septum; LA: left atrium; LV: left ventricle; RA: right atrium; RV: right ventricle

**Figure 4:**
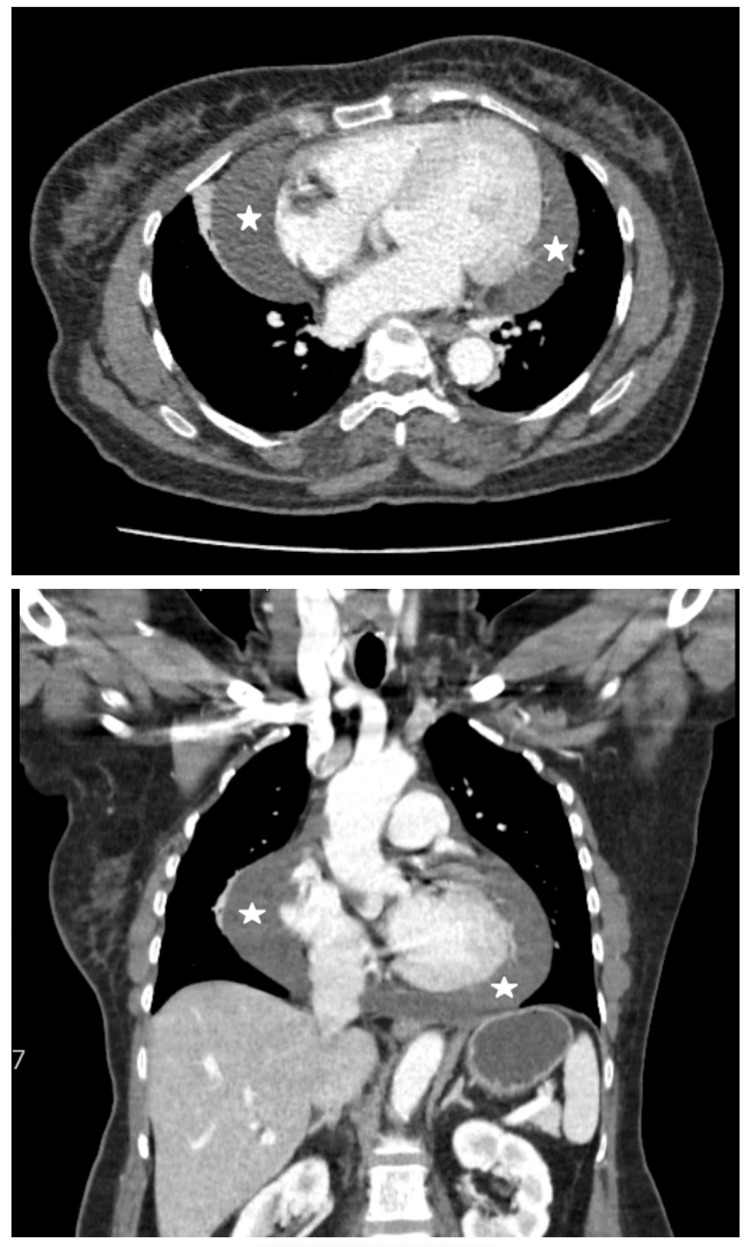
CT of the thorax revealing a large pericardial effusion, as depicted by the white star

Bedside POCUS portrayed a massive pericardial effusion (Figure 3) with mitral valve E-point septal separation of 10 mm, indicating mild to moderately reduced ejection fraction and inferior vena cava (IVC) measuring 1.4 cm with more than 50% collapsibility. There was no evidence of right atrial (RA) systolic collapse or right ventricular (RV) diastolic collapse, which are the main hallmarks of cardiac tamponade. CT thorax revealed cardiomegaly with massive pericardial effusion measuring 29 mm in maximum thickness adjacent to the right atrium (Figure 4). Apart from the atrophic thyroid gland, there were no lung nodules or cavitating lesions. 

Laboratory investigations showed raised thyroid-stimulating hormone (TSH), low free thyroxine (T4), high creatine phosphokinase, raised lactate dehydrogenase, and a deranged lipid profile with otherwise normal N-terminal prohormone of brain natriuretic peptide. Table 1 depicts the relevant laboratory investigations.

**Table 1:** Laboratory investigations HDL: high-density lipoprotein; LDL: low-density lipoprotein; NT-proBNP: N-terminal prohormone of brain natriuretic peptide; T4: free thyroxine; TSH: thyroid-stimulating hormone

Laboratory investigations	Results (at diagnosis)	Normal range
Hemoglobin (g/dL)	12.1	12-15
White blood cells (× 10^3^/µL)	5.5	4-10
Platelet (× 10^9^/L)	186	150-410
Sodium (mmol/L)	132	135-145
Potassium (mmol/L)	3.6	3.3-5.1
Urea (mmol/L)	3.3	1.7-8.3
Creatinine (µmol/L)	103	50-98
TSH (mIU/mL)	55.3	0.27-4.2
T4 (pmol/L)	1.2	12.3-22
Creatine phosphokinase (U/L)	4,100	24-195
Lactate dehydrogenase (U/L)	850	230-460
Total cholesterol (mmol/L)	5.9	<5.2
LDL cholesterol (mmol/L)	2.8	<2.6
HDL cholesterol (mmol/L)	2.2	1.03-4
Triglyceride (mmol/L)	1.9	<1.7
NT-proBNP (pg/mL)	102	<125

**Figure 5:**
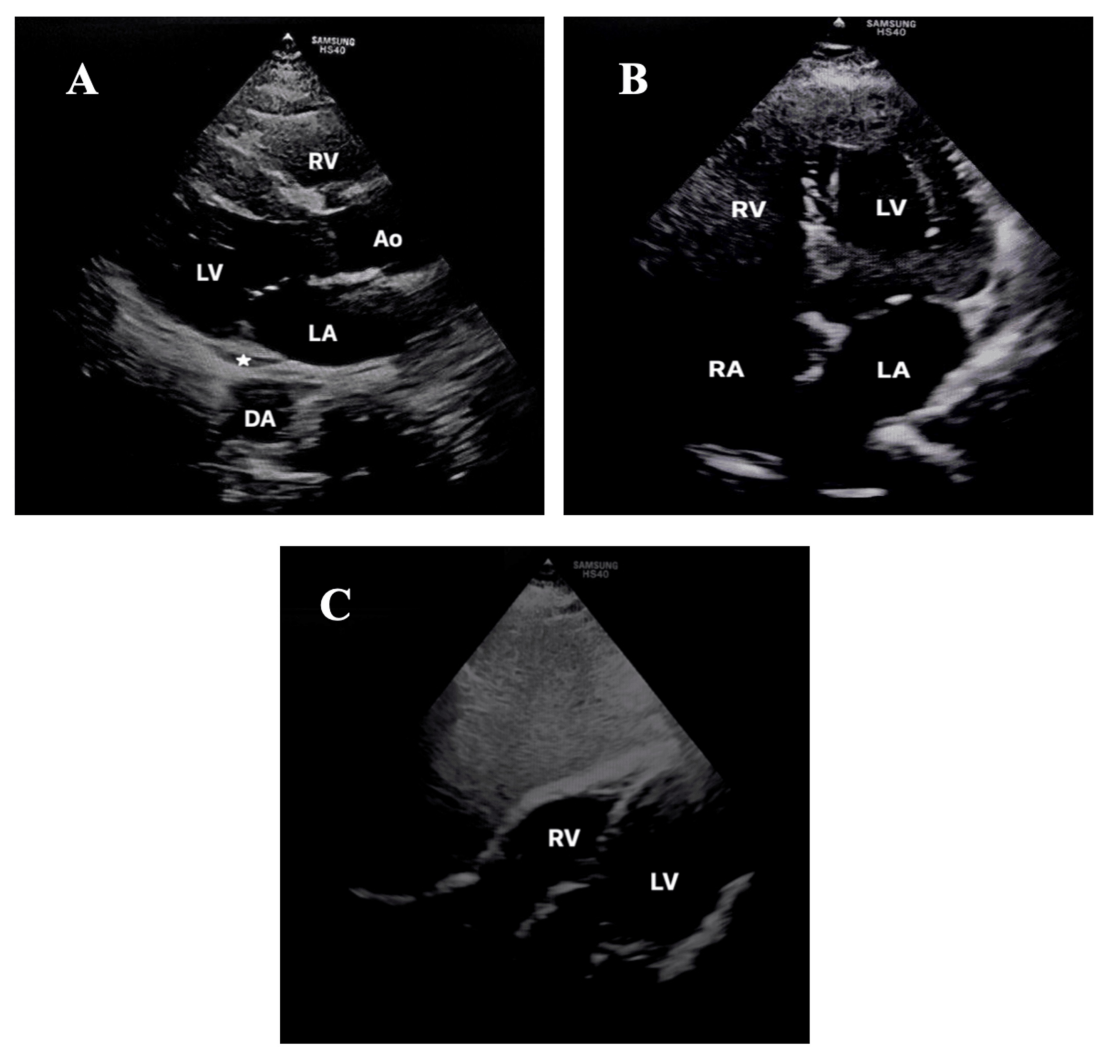
Repeated echocardiogram four months after levothyroxine treatment revealing a significant reduction of pericardial effusion, as depicted by the white star: (A) Parasternal long axis; (B) Apical four-chamber view; (C) Subcostal view Ao: aorta; DA: descending aorta; LA: left atrium; LV: left ventricle; RA: right atrium; RV: right ventricle

**Figure 6:**
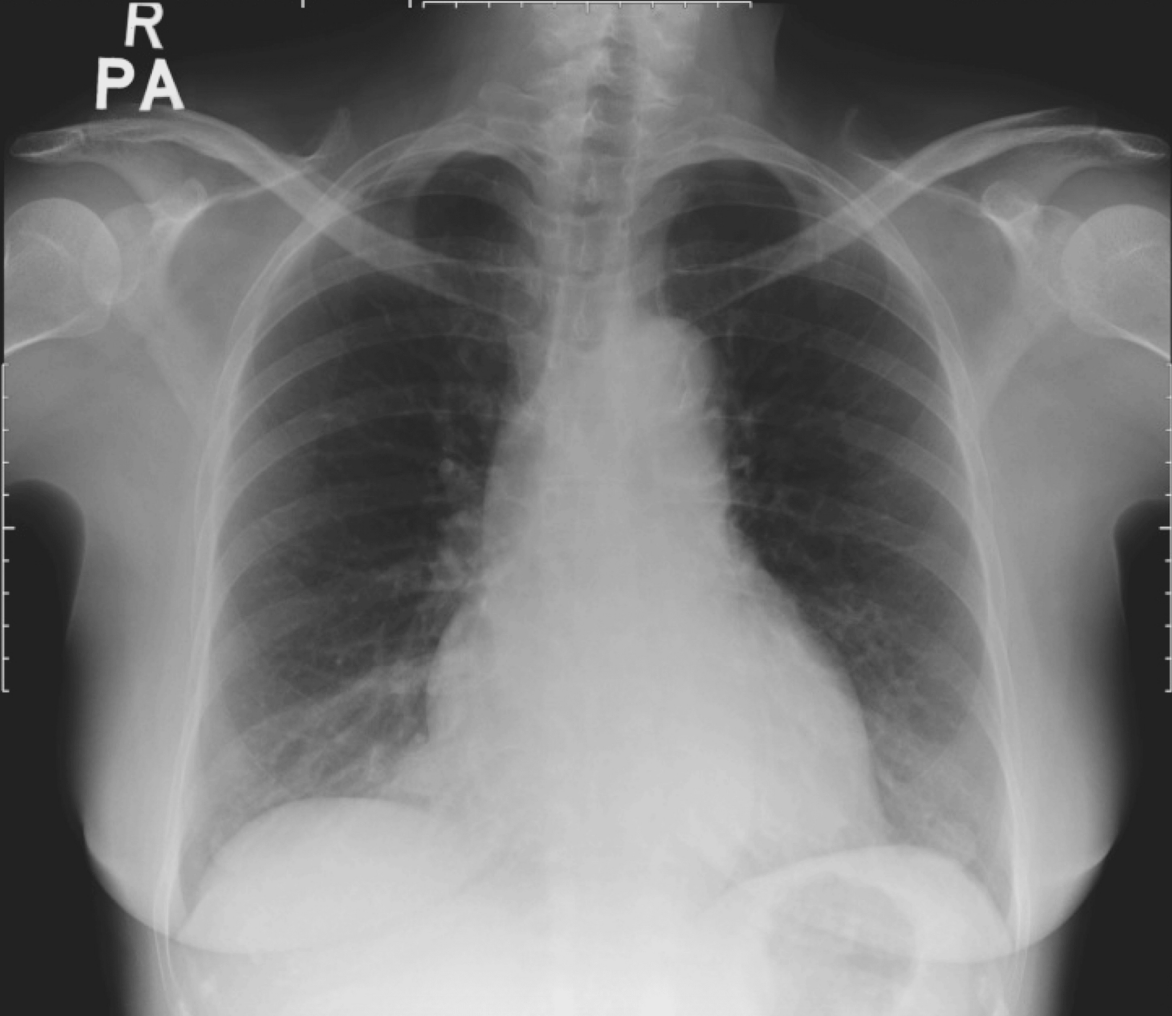
Repeated CXR four months after levothyroxine treatment revealing a significant reduction of cardiac diameter CXR: chest X-ray

As she was hemodynamically stable and exhibited no POCUS features indicative of cardiac tamponade, pericardiocentesis was not performed. Thyroid hormone replacement therapy with levothyroxine 100 mcg daily (1.6 mcg/kg of her body weight of 61 kg daily) was initiated. Table 2 shows the normalization of TSH and free T4 assays two months following treatment. The pericardial effusion gradually subsided during the surveillance echocardiogram four months later (Figure 5). The improvement of the cardiothoracic ratio is evident on the repeat CXR as well (Figure 6).

**Table 2:** Trend of thyroid function tests after treatment with levothyroxine T4: free thyroxine; TSH: thyroid-stimulating hormone

Blood investigations	Admission	Two months post-treatment with levothyroxine	Normal range
TSH (mIU/mL)	55.3	1.09	0.27-4.2
T4 (pmol/L)	1.2	13.8	12.3-22

## Discussion

The use of ultrasound in the diagnosis and evaluation of pericardial disease was first described in 1995 by Edler [6]. POCUS provides a rapid, easily accessible, noninvasive assessment of the pericardium as well as a comprehensive appraisal of the hemodynamic instability in pericardial effusion. A structured cardiac POCUS encompasses the following: localization of the effusion (global or localized); quantification of the effusion (mild <10 mm, moderate 10-20 mm, or severe >20 mm); qualitative assessment of the effusion; and most importantly, analysis of hemodynamic compromise. It is crucial to evaluate pathologies such as pericardial effusion using multiple standard POCUS views. Relying solely on a single POCUS view may introduce errors or artifacts. Incorporating data from the four standard cardiac POCUS views (subcostal, four-chamber, parasternal long and short axis) helps to eliminate the possibility of artifacts, resulting in a more precise and dependable diagnosis of the pathology [7].

**Figure 7:**
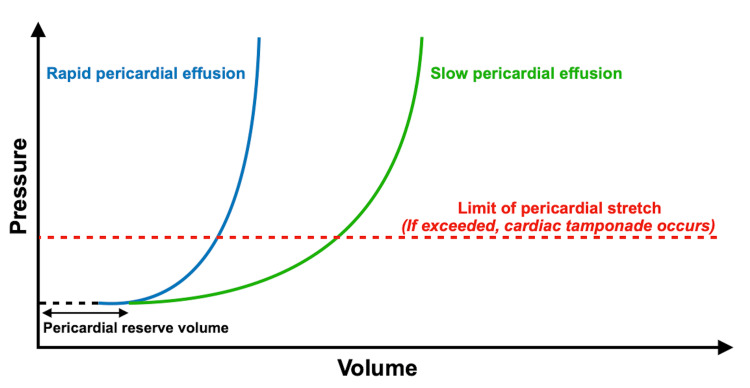
Pressure-volume curve in cardiac tamponade physiology The pericardial space can accommodate the initial increment of pericardial fluid without a significant increase in pressure until the limit of the pericardial reserve volume is reached. Blue curve: In rapidly increasing pericardial effusion, the limit of pericardial stretch is quickly exceeded, resulting in a steep rise in pressure even with small fluid volumes. Green curve: In slowly accumulating pericardial effusion, the pericardium can accommodate larger volumes before reaching a hemodynamic compromise. When the limit of pericardial stretch is exceeded, even small increments in pericardial volume may trigger cardiac tamponade. Image source: Pérez-Casares et al. (2017) [7]; Creative Commons Attribution License (CC BY)

Therefore, in this case, using a variety of cardiac POCUS views helps in determining the magnitude of the pericardial effusion and identifying any features of cardiac tamponade. Although the volume of the pericardial effusion in her case is large, it has no features of cardiac tamponade. The volume of pericardial effusion does not necessarily correspond with clinical symptoms or disease severity on a physiological level. The rationale for this is the fact that parietal pericardium compliance exhibits a nonlinear relationship (Figure 7). Rapid onset and small effusion lead to cardiac tamponade faster in comparison to slowly accumulating pericardial effusion, allowing the pericardium to stretch and reach the critical threshold with a larger volume of effusion [8].

In her case, the discovery of massive pericardial effusion on POCUS in the absence of hemodynamic instability signifies the gradual accumulation of pericardial effusion, which is common in hypothyroidism-associated pericardial effusion. However, in cases of very massive pericardial effusion, some of the patients will present with cardiac tamponade as the first sign of presentation. It is estimated that approximately 2-5% of cardiac tamponade cases could be attributed to hypothyroidism [9].

POCUS is a useful diagnostic tool in the detection of cardiac tamponade. In cardiac tamponade with substantial hemodynamic instability, the increase in intrapericardial pressure may result in the collapse of the RA and RV. RA collapse usually precedes RV collapse during the progression of the cardiac tamponade. RA collapse usually occurs during systole, and the duration of RA collapse longer than one-third of the cardiac cycle has a nearly 100% sensitivity and specificity in cardiac tamponade [10]. RV collapse is commonly observed during diastole, with a sensitivity of 93% but a specificity of 100% for cardiac tamponade [11]. Another important sign is IVC plethora, which indicates dilatation of the IVC (more than 2 cm in adults) with less than 50% collapsibility during inspiration. Although not highly specific, it is a highly sensitive indicator with a 97% sensitivity for cardiac tamponade [12]. All of these cardiac POCUS features can be easily examined during emergency scenarios before embarking on the decision of pericardiocentesis.

## Conclusions

In this case, the initial nonspecific presentation may mimic other medical conditions, such as hypertensive crisis or cerebrovascular events. However, early implementation of bedside POCUS and meticulous review of the patient’s history of thyroid disorders post-RAI in conjunction with her abnormal thyroid function test led to a prompt diagnosis of hypothyroidism-associated pericardial effusion. The cornerstone of the management of hypothyroidism-induced pericardial disease relies heavily on a high index of clinical suspicion among clinicians and early POCUS assessments. Pericardial effusion can be reversible with early initiation of adequate thyroid replacement therapy, thereby averting the fatal complications of cardiac tamponade.
